# Polymorphism and methylation of the *MC4R* gene in obese and non-obese dogs

**DOI:** 10.1007/s11033-017-4114-3

**Published:** 2017-07-28

**Authors:** Monika Mankowska, Joanna Nowacka-Woszuk, Aneta Graczyk, Paulina Ciazynska, Monika Stachowiak, Marek Switonski

**Affiliations:** Department of Genetics and Animal Breeding, Poznan University of Life Sciences, 60-637 Poznan, Poland

**Keywords:** *MC4R*, Obesity, Body weight, Methylation, Labrador Retriever, Golden Retriever, Beagle

## Abstract

The dog is considered to be a useful biomedical model for human diseases and disorders, including obesity. One of the numerous genes associated with human polygenic obesity is *MC4R*, encoding the melanocortin 4 receptor. The aim of our study was to analyze polymorphisms and methylation of the canine *MC4R* in relation to adiposity. Altogether 270 dogs representing four breeds predisposed to obesity: Labrador Retriever (n = 187), Golden Retriever (n = 38), Beagle (n = 28) and Cocker Spaniel (n = 17), were studied. The dogs were classified into three groups: lean, overweight and obese, according to the 5-point Body Condition Score (BCS) scale. In the cohort of Labradors a complete phenotypic data (age, sex, neutering status, body weight and BCS) were collected for 127 dogs. The entire coding sequence as well as 5′ and 3′-flanking regions of the studied gene were sequenced and six polymorphic sites were reported. Genotype frequencies differed considerably between breeds and Labrador Retrievers appeared to be the less polymorphic. Moreover, distribution of some polymorphic variants differed significantly (P < 0.05) between small cohorts with diverse BCS in Golden Retrievers (c.777T>C, c.868C>T and c.*33C>G) and Beagles (c.-435T>C and c.637G>T). On the contrary, in Labradors no association between the studied polymorphisms and BCS or body weight was observed. Methylation analysis, using bisulfite DNA conversion followed by Sanger sequencing, was carried out for 12 dogs with BCS = 3 and 12 dogs with BCS = 5. Two intragenic CpG islands, containing 19 cytosines, were analyzed and the methylation profile did not differ significantly between lean and obese animals. We conclude that an association of the *MC4R* gene polymorphism with dog obesity or body weight is unlikely, in spite of the fact that some associations were found in small cohorts of Beagles and Golden Retrievers. Also methylation level of this gene is not related with dog adiposity.

**Electronic supplementary material:**

The online version of this article (doi:10.1007/s11033-017-4114-3) contains supplementary material, which is available to authorized users.

## Introduction

Since dogs share the same environment and lifestyle with humans they are also susceptible to the same civilization-related diseases and among them obesity has become an emerging health problem in both species. An overall obesity rate in dogs ranges from 25.0% in Australia to 59.3% in UK [1–5]. It may be underestimated, because in certain breeds (e.g. Labrador Retriever) individuals considered as being in a good body condition are in fact overweight [6]. Except for obvious positive outcomes for dogs’ welfare itself, the reason to study obesity in this species is due to its model role for human diseases and their therapies [7].

The melanocortin receptor gene family consists of five members and one of them, *MC4R*, has attracted special attention due to its crucial role in energy homeostasis [8]. Numerous reports show that some mutations in this gene cause monogenic obesity in humans [9]. Moreover, a meta-analysis revealed an association of several DNA variants located within the *MC4R* gene or in its vicinity with the accumulation of adipose tissue [10]. Some variants, p.Val103Ile and p.Ile251Leu, play a protective role [9], while rs17782313 (SNP C/T), located almost 188 kb upstream from this gene, predisposes to polygenic obesity [11] and is associated both with eating behavior [12] and physical activity [13].

Until now 19 polymorphic sites were reported in the canine *MC4R* gene or in its vicinity (NCBI SNP database http://www.ncbi.nlm.nih.gov/snp/). Some of them were analyzed for an association with obesity, body weight or measures, but the obtained results were ambiguous, possibly due to small cohorts analyzed [14–16].

Epigenetic mechanisms, e.g. DNA methylation, may also play an important role in pathogenesis of some diseases, including obesity and type 2 diabetes [17, 18]. It was shown that high-fat diet alters methylation pattern of the mice *Mc4r* gene [19]. Until now no reports are available on the methylation profile of the canine *MC4R* gene. Taking the above into consideration we aimed to search for polymorphisms in coding and flanking sequences of the *MC4R* gene, as well as analyze its methylation level in obese and non-obese dogs.

## Materials and methods

Blood samples from 270 dogs (130 males and 140 females, among them 181 intact and 89 neutered) were collected. Dogs had no record of any medical problems and were sampled during standard veterinarian visit with the consent of their owners. The studied cohort was composed of dogs with well-known obesity susceptibility: Labrador Retrievers (n = 187), Golden Retrievers (n = 38), Beagles (n = 28) and Cocker Spaniels (n = 17). All individuals were annotated to an appropriate Body Condition Score (BCS) group, based on visual and palpable assessment. For the purpose of our study we have chosen a 5-point BCS scale: 3—normal (n = 108), 4—overweight (n = 97) and 5—obese (n = 65). However, complete phenotypic data including: sex, age, neutered status, body weight and BCS could be collected for: 127 Labrador Retrievers (Supplementary Table S1), 24 Golden Retrievers, 7 Beagles and 6 Cocker Spaniels. Thus, detailed analysis of association between *MC4R* polymorphism and BCS, as well as body weight was performed for Labradors, only.

### DNA sequencing

DNA from blood was extracted using the BloodMini Isolation Kit (A&A Biotechnology) protocol. For further analysis a coding sequence of the *MC4R* gene (999 bp, GenBank NC_006583.3) with adjacent 5′ and 3′ regions was amplified in four PCRs, covering over 2000 bp. Amplification was performed with primers designed by Primer3 (http://bioinfo.ut.ee/primer3-0.4.0/; Supplementary Table S2) applying to the following conditions—initial denaturation (94 °C; 5 min), 35 cycles of denaturation (94 °C; 40 s), annealing (respective temperatures in Supplementary Table S2; 30 s) and elongation (72 °C; 40 s); the final elongation (72 °C; 10 min). Prior to sequencing PCR products were purified with Alkaline phosphatase and Exonuclease I (Thermo Fisher Scientific), amplified with the BigDye Terminator v3.1 Kit (Thermo Fisher Scientific) and filtrated on Sephadex G50 (Sigma). Sequencing was performed on a Genetic Analyzer 3130 capillary sequencer (Applied Biosystems).

### Methylation analysis

Based on the reference genomic sequence (NC_006583.3) the in silico studies of the *MC4R* gene and an upstream region of 1000 bp was inspected in terms of the CpG islands (CGI) existence using the CpGPlot software (http://www.ebi.ac.uk/Tools/seqstats/emboss_cpgplot/). Two short CGIs (159 and 146 bp in length) were found in the coding sequence of the gene and the PCR primers were designed using the MethPrimer software (http://www.urogene.org/cgi-bin/methprimer/methprimer.cgi): 5′F: TGAGTTTTTTGGTAAAGGTTATTT; 5′R: CTACAATTAAAAACAAACTACAAATC. The amplicon length was 350 bp and overlapped with 19 CG dinucleotides (Supplementary Fig. S1). The analysis was performed for 24 animals classified as BCS = 3 (n = 12) and BCS = 5 (n = 12), representing three breeds: Labrador Retriever (n = 12), Golden Retriever (n = 6) and Beagle (n = 6).

After extraction from blood (Epicentre), 1 µg of DNA was bisulfite converted according to the EZ DNA Methylation kit (ZymoResearch) protocol and amplified with touch-down PCR (for the initial 8 cycles the annealing temperature was decreased by 0.5 °C per cycle in a range from 60 to 56 °C, while for the next 32 cycles primers were annealed at 56 °C). The PCR products were cloned into the pGEM T-Easy Vector (Promega) following transformation of DH5α competent cells (Invitrogen) by heat shock and cultured overnight at 37 °C on agar plates with an addition of IPTG, X-gal and ampicillin. For each individual, at least 12 positive (white) colonies were picked and amplified with the Illustra ThempliPhi Amplification Kit (GE Healthcare). In order to quantify the methylation level the clones were sequenced using the Big Dye v1.1 Termination Kit (Thermo Fisher Scientific) on a Genetic Analyzer 3130.

### Statistical analysis

Results of DNA sequencing were analyzed with the use of the Lasergene SeqMan software (DNASTAR). Allele and genotype frequencies along with odds ratio values (https://www.medcalc.org/calc/odds_ratio.php) were calculated. The HaploView software [20] was used for haplotype analysis. Additionally, for the largest group of Labrador Retrievers (n = 127) with the complete phenotypic data set a further statistical association analysis was carried out. The genotype, age, sex and neutered status were used as independent variables affecting dependent variable—body weight or BCS. Generalized linear model (GLM) procedure from SAS Enterprise 6.1 package [21] was applied in order to evaluate effects on body weight. For BCS a logistic regression (LOGIT) model was used. The methylation results were analyzed using the QUMA software (http://quma.cdb.riken.jp). The statistical analysis of the methylation level between BCS = 3 and BCS = 5 dogs was performed with the use of the SigmaStat 3.5 software (Systat Software).

## Results

### Sequence analysis

DNA sequencing of the entire coding sequence of *MC4R* (999 bp), as well as the 5′-flanking region (541 bp) and the 3′-flanking region (518 bp), revealed six known polymorphic sites. Three of them were found in the coding sequence at positions: c.637G>T (rs852614811), c.777T>C (rs851987283) and c.868C>T (rs851062983). Among them only c.637G>T was a nonsynonymous substitution (p.Val213Phe). Two polymorphisms were identified in the 3′-flanking region: c.*33C>G (rs851539399) and c.*227C>T [14] and one in the 5′-flanking region, at position c.-435T>C (rs852471376).

The alignment of canine 5′ and 3′-flanking sequences to other species (the human, mouse, pig and cattle) led us to the assumption that both polymorphic sites downstream of the coding sequence reside in the 3′UTR. Searching for target sequences for miRNA in this region (miRSearch V3.0; http://www.targetscan.org/) gave a negative result. According to the MatInspector software the appearance of c.-435T>C polymorphism introduces a consensus sequence for the cell cycle-dependent element (CDE) for the CDF-1 factor, which plays a role in cell cycle regulation [22].

Frequency of the detected variants showed considerable differences between breeds. Detailed information on variant frequencies is given in Supplementary Table S3. The lowest level of polymorphism was observed in Labrador Retrievers, where 4 out of 6 detected SNPs had a minor allele frequency (MAF) below 0.05 (Table 1).

**Table 1:** Frequency of reference variants (according to NC_006583.3) at 6 SNP sites

SNP	Reference variant	Beagle (n = 28)	Cocker Spaniel (n = 17)	Golden Retriever (n = 38)	Labrador Retriever (n = 187)
c.-435T>C	T	0.67	0.91	0.89	0.22
c.637G>T	G	0.59	0.76	0.72	0.96
c.777T>C	T	0.20	0.56	0.13	0.00
c.868C>T	C	1.00	1.00	0.61	1.00
c.*33C>G	C	0.20	0.56	0.13	0.00
c.*227C>T	C	1.00	0.91	0.96	0.24

Haplotype analysis across breeds revealed the presence of 14 haplotypes and their frequency also varied between breeds (Table 2). In Labrador Retrievers the CGCCGT haplotype was predominant: 0.78 (BCS = 3) and 0.73 (BCS = 4 or 5). Interestingly, this haplotype was rarely observed (0.03) in Golden Retrievers with BCS = 3 and was not found in dogs with BCS = 4 or 5. This variant was present neither in Cocker Spaniels nor Beagles.

**Table 2:** Haplotype frequencies in the studied breeds

Haplotype	Beagle		Cocker Spaniel		Golden Retriever		Labrador Retriever	
	BCS = 3	BCS = 4 or 5	BCS = 3	BCS = 4 or 5	BCS = 3	BCS = 4 or 5	BCS = 3	BCS = 4 or 5
CGCCGC	0.26	0.31	0.04	–	–	–	0.02	0.03
CGCCGT	–	–	–	–	0.03	–	0.78	0.73
TGCCGC	0.11	0.11	0.11	0.12	0.20	0.15	–	0.18
TGCCGT	–	–	0.08	–	–	0.02	–	0.02
CGCTGC	–	–	–	–	–	0.07	–	–
TGCTGC	–	–	–	–	0.27	0.41	0.15	–
CGTCCC	–	–	–	0.12	0.10	–	–	–
CGTCCT	–	–	–	0.13	–	–	–	–
TGTCCC	0.10	0.31	0.50	0.50	0.10	0.07	–	–
TGTCCT	–	–	–	–	0.03	–	–	–
CTCCGC	0.04	–	–	–	0.03	–	–	–
CTCCGT	0.04	–	–	–	–	–	–	–
TTCCGC	0.42	0.27	0.27	0.13	0.24	0.28	0.05	0.04
TTCCGT	0.03	–	–	–	–	–	–	–

The order of SNPs is as follows: c.-435 T>C; c.637G>T; c.777T>C; c.868C>T; c.*33C>G and c.*227C>T

The odds ratio (OR) test, applied for comparison of polymorphic variants distribution in dogs with different BCS revealed significant relationships for 3 SNPs in Golden Retrievers and 2 SNPs in Beagles (Table 3). The most pronounced results were observed in Beagles for c.-435T>C and c.637G>T (p.Val213Phe) polymorphisms when lean (BCS = 3) and obese dogs (BCS = 5), as well as lean and overweight/obese dogs (BCS = 4 or 5) were compared. On the contrary, in the biggest cohort of Labrador Retrievers (n = 187) no association was found.

**Table 3:** Significant (P < 0.05) associations between SNPs and BCS, revealed by the odds ratio (OR) test

SNP and compared groups in terms of BCS	Breed			
	Golden Retriever (n = 38)		Beagle (n = 28)	
	OR	P	OR	P
c.-435T>C		ns		
3 versus 5			5.000	0.0275
3 versus 4 and 5			3.750	0.0109
c.637G>T (p.Val213Phe)		ns		
3 versus 4			6.8571	0.0230
3 versus 4 and 5			3.1020	0.0486
c.777T>C				ns
3 versus 4 and 5	0.2292	0.0456		
c.868C>T				ns
3 versus 5	0.2909	0.0496		
c.*33C>G				ns
3 versus 4 and 5	0.2292	0.0456		

*ns* non-significant

The GLM procedure applied for Labradors revealed significant effects of age and sex on body weight (P < 0.0001). Females were lighter than males with the estimated effect of 3.9 kg and each month estimated effect was +0.07 kg. Neither neutering nor genotypes in *MC4R* gene influenced body weight. In the LOGIT model statistically significant effects on BCS in Labradors were registered for age (P < 0.0001) and neutering (P = 0.036), while effects of sex and genotype were not significant.

### Methylation analysis

**Fig. 1:**
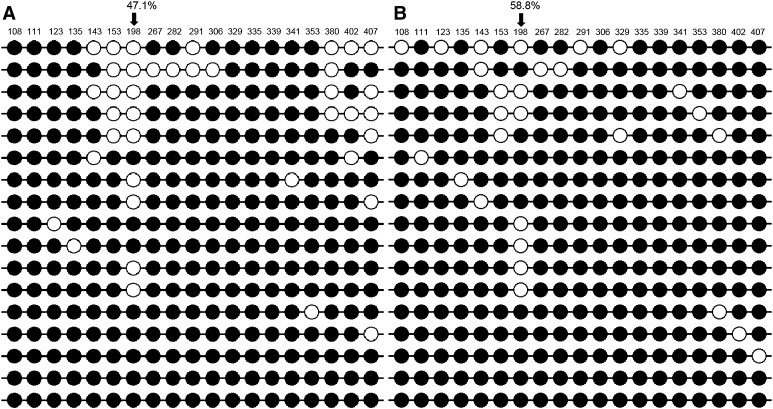
An example of methylation patterns in two Labrador Retrievers, **a** BCS = 3 and **b** BCS = 5. *Black circle* cytosine methylated, *white circle* cytosine non-methylated. *Each line* represents particular clone sequencing results. An *arrow* indicates the cytosine with the lowest methylation (% given above)

The analyzed amplicon (350 bp) contained 19 CG dinucleotides (Supplementary Fig. S1) and a majority of them showed high (over 80%) cytosine methylation. The only exception (Fig. 1) was observed at position c.198 (numbered according to the *MC4R* gene coding sequence NCBI accession no. NC_006583.3), where the cytosine methylation rate was lower in all analyzed breeds. This cytosine is located between two CGIs. The detailed results are presented in Supplementary Table S4. The statistical analysis (average % of methylation for each cytosine) showed no significant differences either in terms of methylation status between BCS = 3 and BCS = 5 Labrador Retrievers or across all breeds.

## Discussion

Association of DNA polymorphisms with dogs’ predisposition to obesity is still poorly recognized. Until now such results have been described for the *TNF* [23], *POMC* [14, 24], *MC4R* [16] and *GPR120* [25] genes. However, the observations for the *MC4R* gene are ambiguous.

In this study we sequenced the *MC4R* gene of 270 dogs and six previously reported SNPs were found, which segregated in 14 haplotypes. We found that the lowest level of polymorphism was observed in Labrador Retrievers. These results show that the extent of the *MC4R* polymorphism is much lower than in the human counterpart, for which around 300 variants were reported in the coding, 5′UTR and 3′UTR sequences (NCBI SNP database http://www.ncbi.nlm.nih.gov/snp/).

The association analysis of the detected SNPs with the BCS provided no definite results. None of the SNPs showed an across-breed association. A significant association was observed for SNPs: c.777T>C; c.868C>T; c.*33C>G in Golden Retrievers and c.-435T>C, c.637G>T in Beagles, whereas no association was found in Labrador Retrievers and Cocker Spaniels. The most interesting SNP is a nonsynonymous substitution at position c.637G>T (rs852614811, p.Val213Phe). However, functionality of this substitution is rather doubtful, since it is localized within the transmembrane segment of the encoded protein [26] and does not alter ligand binding and signaling properties of the encoded receptor [27]. Thus, the above mentioned association results should be considered with caution, especially due to a small number of dogs analyzed. In Golden Retrievers a significant association was found for c.777T>C (rs851987283). Lack of the association of this SNP was earlier reported in female Beagle dogs by Zeng et al. [16], who described this substitution as 895C>T. Interestingly, another nonsynonymous substitution c.301A>C, reported by those authors as 420A>C (p.Asn101Thr) and not detected in our study, showed an association with body weight in this breed [16]. The effect of the *MC4R* polymorphism (c.637G>T, c.777T>C, 868C>T and c.*33C>G) on morphometric traits (weight, length and height) of Golden Retrievers (n = 187) was studied by van den Berg et al. [15], who reported no such effect. The above-mentioned reports indicate that the importance of the *MC4R* polymorphism in relation to dogs’ obesity is not clear. Finally, there are no studies showing an association of this gene with body weight or obesity in the Labrador Retriever breed. Interestingly, in this breed such associations were described for two genes: *TNF* [23] and *POMC* [14, 24].

Evaluation of the *MC4R* gene methylation in peripheral blood cells may be considered a controversial task, since its expression is relatively low when compared to the brain. However, the use of a tissue, which is not a major site of expression of a given gene (e.g. *TNF, POMC* and *NPY*) for diagnostic purposes may be efficient [28, 29]. It was also shown that methylation of the human *MC4R*, analyzed in cord blood cells, was associated with triglyceride level in preterm and term infants [30]. Thus, analysis of the *MC4R* methylation in blood cells is justified. Another issue concerns the importance of methylation within a gene body, which has received more attention since it was shown that the methylation level within the gene is much higher than in 5′- and 3′-flanking regions [31]. Moreover, such hypermethylation may positively correlate with transcription level, in contrast to repression of the expression due to promoter methylation. However, some studies have shown a negative correlation between intragenic methylation and transcriptional elongation [32] and splicing [33]. Moreover, there are also observations that DNA methylation interacts with histone modifications and intragenic methylation contributes to proper transcript elongation by removing repressive epigenetic marks (H3K27me3) and recruiting H3K36me3 marks, which are accumulated in actively transcribed units [32]. With regard to the *MC4R* gene it was shown that methylation of its coding region in mice is influenced by diet [19]. The authors analyzed brain tissue and showed that a high-fat diet caused a decrease in methylation of the CGIs near the transcription start site.

Our in silico analysis of the 1000 bp fragment upstream of the *MC4R* start codon showed no CGI, while in the coding sequence two CGIs were found. The level of individual cytosine methylation was high and did not differ between lean and obese dogs in the studied breeds. Thus, the methylation profile of the *MC4R* gene analyzed in blood cannot be considered as an epigenetic marker of adiposity.

We conclude that in contrast to the human it is rather unlikely that the *MC4R* gene is significantly associated with predisposition to obesity across all dog breeds. Also methylation of the *MC4R* gene body is not related with dog obesity.

## Electronic supplementary material

Below is the link to the electronic supplementary material.

Supplementary material 1 (DOC 488 KB)
