# Supplementary material for: Polymorphism and methylation of the MC4R gene in obese and non-obese dogs
Source: Mol Biol Rep. 2017 Jul 28;44(4):333–9. doi: 10.1007/s11033-017-4114-3 (PMC5579139; doi:10.1007/s11033-017-4114-3)
Supplement: Supplementary file 1 — Supplementary material 1 (DOC 488 KB) [file 11033_2017_4114_MOESM1_ESM.doc]

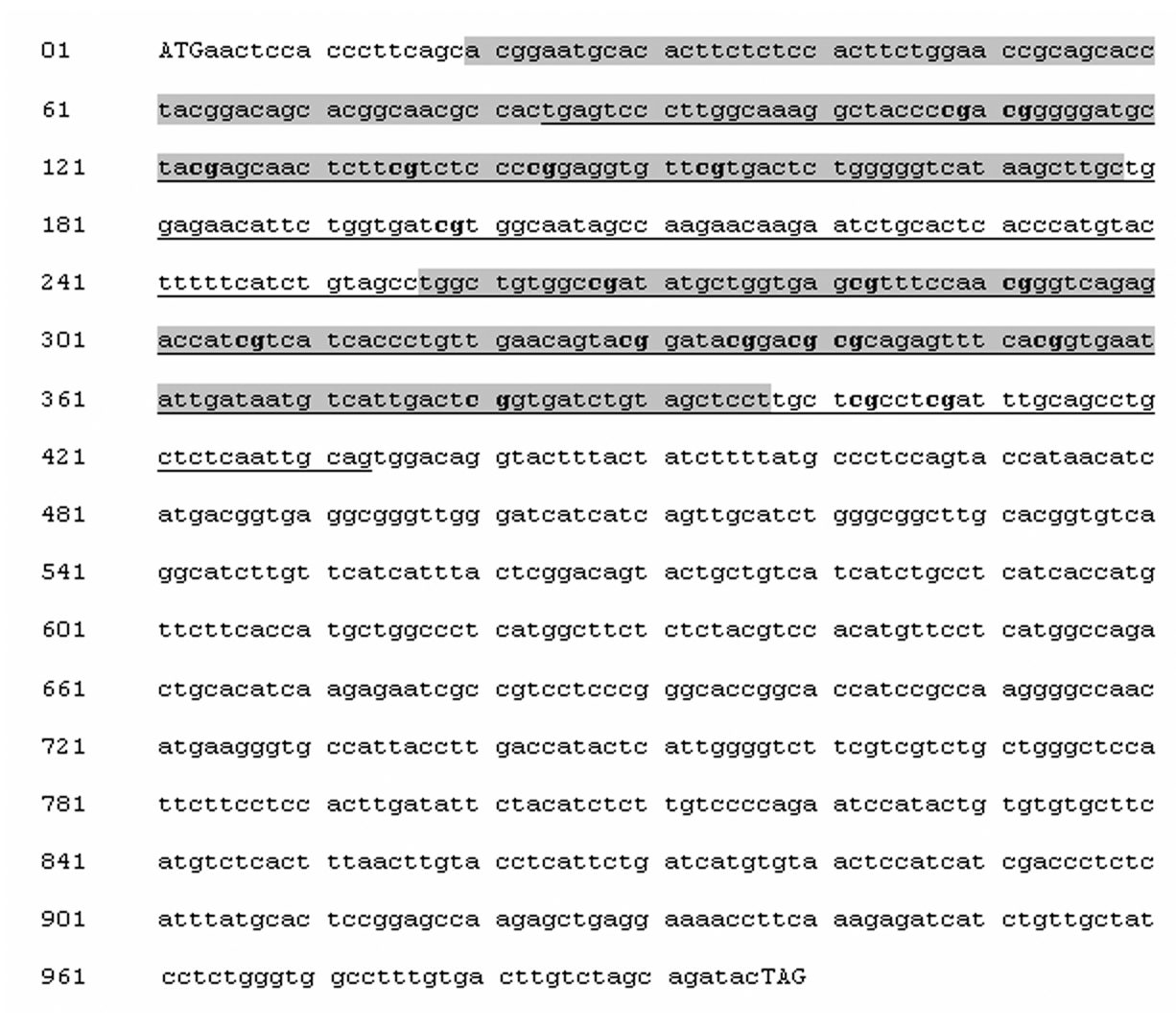


Suppl. Fig. S1. The coding sequence of the *MC4R* gene. In capital letters the START and STOP codons, marked with gray – two CGIs analyzed, underlined – the PCR amplicon used in methylation studies; in bold – CG dinucleotides.

Supplementary Table S1

Characterization of Labrador dogs (n=127) with complete phenotypic data.

| BCS | Females | | | Males | | | Status of neutering |
| --- | --- | --- | --- | --- | --- | --- | --- |
|  | n | Mean age (SD)  [months] | Body weight (SD)  [kg] | n | Mean age (SD)  [months] | Body weight (SD)  [kg] |  |
| 3 | 14 | 20.1 (6.6) | 28.2 (2.7) | 9 | 44.8 (37) | 34.7 (4.4) | intact |
|  | 8 | 67.5 (30.6) | 29.6 (1.7) | 4 | 45 (19.7) | 30.8 (2.6) | neutered |
| 4 | 24 | 43.5 (27.9) | 34.1 (2.6) | 22 | 64.5 (40.6) | 38.3 (3.1) | intact |
|  | 6 | 54 (15.1) | 33.2 (3.1) | 7 | 54.4 (27.8) | 39.7 (2.7) | neutered |
| 5 | 13 | 66.9 (27.8) | 41.1 (3.4) | 12 | 82 (31) | 44.2 (4.4) | intact |
|  | 5 | 87.4 (19.5) | 40.2 (2.5) | 3 | 76 (11.3) | 47.2 (10.6) | neutered |
| Together | 70 | 51.9 (35.7) | 33.7 (5.2) | 57 | 64.2 (36.2) | 39.1 (5.8) |  |

Supplementary Table S2

PCR conditions used in studies of the canine *MC4R* gene.

|  | Amplified region |  | Sequence | Annealing temp. |
| --- | --- | --- | --- | --- |
| 1 | 5' flanking region | F | AGGAGGAGCCACTCTGAACA | 62.0 ^0^C |
| 2 |  | R | TATTGCCACGATCACCAGAA |  |
| 3 | 3' flanking region | F | CCATCATCGACCCTCTCATT | 62.0 ^0^C |
| 4 |  | R | TGCAACAGGGAGAATGAACA |  |
| 5 | CDS part 1 | F | CCAGCTGGATCCTCAGAACT | 63.5 ^0^C |
| 6 |  | R | GCATGGTGAAGAACATGGTG |  |
| 7 | CDS part 2 | F | TCTCAATTGCAGTGGACAGG | 63.5 ^0^C |
| 8 |  | R | CAGCTGTTGTCCAAGCACAC |  |

Supplementary Table S3

Genotype frequencies at 6 SNP sites in the studied dog breeds.

| **Breed** |  | Labrador Retriever | | | | Golden Retriever | | | | Beagle | | | | Cocker Spaniel | | | |
| --- | --- | --- | --- | --- | --- | --- | --- | --- | --- | --- | --- | --- | --- | --- | --- | --- | --- |
| **BCS** |  | 3 | 4 | 5 | all | 3 | 4 | 5 | all | 3 | 4 | 5 | all | 3 | 4 | 5 | all |
| **N** |  | 65 | 74 | 48 | 187 | 15 | 14 | 9 | 38 | 15 | 7 | 6 | 28 | 13 | 2 | 2 | 17 |
| **c.-435T>C** | **TT** | 0.02 | 0.07 | 0.02 | 0.04 | 0.80 | 0.86 | 0.89 | 0.84 | 0.43 | 0.29 | 0.50 | 0.41 | 0.92 | 1.00 | 0.50 | 0.88 |
|  | **TC** | 0.35 | 0.27 | 0.54 | 0.37 | 0.07 | 0.14 | 0.11 | 0.11 | 0.43 | 0.71 | 0.50 | 0.52 | 0.08 | 0.00 | 0.00 | 0.06 |
|  | **CC** | 0.63 | 0.66 | 0.44 | 0.59 | 0.13 | 0.00 | 0.00 | 0.05 | 0.14 | 0.00 | 0.00 | 0.07 | 0.00 | 0.00 | 0.50 | 0.06 |
| **c.637G>T, Val213Phe** | **GG** | 0.89 | 0.95 | 0.92 | 0.92 | 0.60 | 0.57 | 0.44 | 0.55 | 0.27 | 0.71 | 0.33 | 0.39 | 0.54 | 0.50 | 1.00 | 0.59 |
|  | **GT** | 0.11 | 0.05 | 0.06 | 0.07 | 0.27 | 0.36 | 0.44 | 0.34 | 0.40 | 0.29 | 0.50 | 0.39 | 0.38 | 0.50 | 0.00 | 0.35 |
|  | **TT** | 0.00 | 0.00 | 0.02 | 0.01 | 0.13 | 0.07 | 0.11 | 0.11 | 0.33 | 0.00 | 0.17 | 0.21 | 0.08 | 0.00 | 0.00 | 0.06 |
| **c.777T>C** | **TT** | 0.00 | 0.00 | 0.00 | 0.00 | 0.07 | 0.00 | 0.00 | 0.03 | 0.00 | 0.00 | 0.17 | 0.04 | 0.23 | 0.00 | 1.00 | 0.29 |
|  | **TC** | 0.00 | 0.00 | 0.02 | 0.01 | 0.33 | 0.14 | 0.11 | 0.21 | 0.20 | 0.71 | 0.17 | 0.32 | 0.54 | 1.00 | 0.00 | 0.53 |
|  | **CC** | 1.00 | 1.00 | 0.98 | 0.99 | 0.60 | 0.86 | 0.89 | 0.76 | 0.80 | 0.29 | 0.67 | 0.64 | 0.23 | 0.00 | 0.00 | 0.18 |
| **c.868C>T** | **CC** | 1.00 | 1.00 | 1.00 | 1.00 | 0.60 | 0.29 | 0.22 | 0.39 | 1.00 | 1.00 | 1.00 | 1.00 | 1.00 | 1.00 | 1.00 | 1.00 |
|  | **CT** | 0.00 | 0.00 | 0.00 | 0.00 | 0.27 | 0.57 | 0.44 | 0.42 | 0.00 | 0.00 | 0.00 | 0.00 | 0.00 | 0.00 | 0.00 | 0.00 |
|  | **TT** | 0.00 | 0.00 | 0.00 | 0.00 | 0.13 | 0.14 | 0.33 | 0.18 | 0.00 | 0.00 | 0.00 | 0.00 | 0.00 | 0.00 | 0.00 | 0.00 |
| **c.*33C>G** | **CC** | 0.00 | 0.00 | 0.00 | 0.00 | 0.07 | 0.00 | 0.00 | 0.03 | 0.00 | 0.00 | 0.17 | 0.04 | 0.23 | 0.00 | 1.00 | 0.29 |
|  | **CG** | 0.00 | 0.00 | 0.02 | 0.01 | 0.33 | 0.14 | 0.11 | 0.21 | 0.20 | 0.71 | 0.17 | 0.32 | 0.54 | 1.00 | 0.00 | 0.53 |
|  | **GG** | 1.00 | 1.00 | 0.98 | 0.99 | 0.60 | 0.86 | 0.89 | 0.76 | 0.80 | 0.29 | 0.67 | 0.64 | 0.23 | 0.00 | 0.00 | 0.18 |
| **c.*227C>T** | **CC** | 0.03 | 0.07 | 0.08 | 0.06 | 0.87 | 0.92 | 1.00 | 0.92 | 0.93 | 1.00 | 1.00 | 1.00 | 0.92 | 1.00 | 0.50 | 0.88 |
|  | **CT** | 0.38 | 0.25 | 0.50 | 0.36 | 0.13 | 0.08 | 0.00 | 0.08 | 0.00 | 0.00 | 0.00 | 0.00 | 0.00 | 0.00 | 0.50 | 0.06 |
|  | **TT** | 0.58 | 0.68 | 0.42 | 0.58 | 0.00 | 0.00 | 0.00 | 0.00 | 0.07 | 0.00 | 0.00 | 0.04 | 0.08 | 0.00 | 0.00 | 0.06 |

Supplementary Table S4

An average methylation level (%) of each cytosine, studied in relation to breed and BCS.

| CG position | Methylation level (% ± Std Dev.) | | | | | |
| --- | --- | --- | --- | --- | --- | --- |
|  | Labrador BCS=3 (n=6) | Labrador BCS=5 (n=6) | Golden Retriever BCS=3 (n=3) | Golden Retriever BCS=5 (n=3) | Beagle BCS=3 (n=3) | Beagle BCS=5 (n=3) |
| 108 | 95.2 ± 8.6 | 96.6 ± 3.8 | 94.4 ± 9.6 | 84.4 ± 7.7 | 95.2 ± 8.3 | 97.6 ± 4.1 |
| 111 | 100.0 ± 0.0 | 97.6 ± 3.7 | 94.9 ± 4.5 | 95.5 ± 3.9 | 95.3 ± 4.1 | 97.6 ± 4.1 |
| 123 | 90.3 ± 6.1 | 92.9 ± 6.4 | 94.5 ± 4.8 | 93.3 ± 6.7 | 95.1 ± 4.3 | 97.6 ± 4.1 |
| 135 | 86.1 ± 15.4 | 90.5 ± 5.9 | 92.1 ± 8.4 | 88.9 ± 3.8 | 87.7 ± 4.5 | 93.3 ± 6.7 |
| 143 | 86.7 ± 11.5 | 91.6 ± 7.9 | 94.4 ± 9.6 | 93.3 ± 6.7 | 77.5 ± 7.8 | 93.2 ± 0.2 |
| 153 | 82.6 ± 7.2 | 77.1 ± 10.5 | 94.4 ± 9.6 | 86.6 ± 11.5 | 80.4 ± 4.7 | 86.0 ± 14.3 |
| 198 | 62.2 ± 15.9 | 48.5 ± 19.9 | 63.1 ± 4.3 | 64.5 ± 23.4 | 61.2 ± 22.6 | 61.2 ± 10.6 |
| 267 | 87.5 ± 6.8 | 88.1 ± 6.1 | 84.9 ± 11.7 | 84.5 ± 3.9 | 97.6 ± 4.1 | 84.3 ± 7.4 |
| 282 | 95.3 ± 3.7 | 96.5 ± 4.0 | 97.2 ± 4.8 | 91.1 ± 3.8 | 90.3 ± 10.8 | 97.8 ± 3.9 |
| 291 | 90.4 ± 8.9 | 89.9 ± 10.7 | 92.5 ± 7.2 | 88.9 ± 19.2 | 92.5 ± 7.7 | 93.0 ± 7.2 |
| 306 | 92.6 ± 4.9 | 92.9 ± 6.4 | 90.1 ± 10.8 | 93.3 ± 0.0 | 87.7 ± 4.5 | 100.0 ± 0.0 |
| 329 | 96.3 ± 6.4 | 94.1 ± 4.8 | 91.7 ± 8.4 | 91.5 ± 15.4 | 100.0 ± 0.0 | 100.0 ± 0.0 |
| 335 | 96.1 ± 4.3 | 96.3 ± 4.1 | 100.0 ± 0.0 | 100.0 ± 0.0 | 92.5 ± 7.7 | 97.8 ± 3.9 |
| 339 | 95.9 ± 6.9 | 100.0 ± 0.0 | 94.9 ± 4.5 | 93.3 ± 6.7 | 97.6 ± 4.1 | 97.8 ± 3.9 |
| 341 | 96.2 ± 6.8 | 96.7 ± 3.8 | 100.0 ± 0.0 | 100.0 ± 0.0 | 97.6 ± 4.1 | 100.0 ± 0.0 |
| 353 | 99.0 ± 2.4 | 94.3 ± 5.4 | 97.2 ± 4.8 | 97.8 ± 3.9 | 92.9 ± 7.2 | 97.8 ± 3.9 |
| 380 | 89.8 ± 8.1 | 87.7 ± 9.4 | 94.9 ± 4.5 | 86.7 ± 6.7 | 88.1 ± 10.9 | 83.8 ± 11.2 |
| 402 | 85.4 ± 9.2 | 85.3 ± 10.0 | 84.9 ± 14.4 | 84.4 ± 7.7 | 92.5 ± 7.7 | 86.5 ± 6.5 |
| 407 | 76.3 ± 14.4 | 86.9 ± 18.3 | 79.0 ± 4.2 | 80.0 ± 6.7 | 83.1 ± 10.7 | 81.9 ± 3.3 |
